# Sensitising PDAC to Gemcitabine by Suppressing NF-κB Pathway and Enhancing Apoptosis

**DOI:** 10.3390/ph19020243

**Published:** 2026-01-30

**Authors:** Enhui Jin, Maria Rita Gil da Silva Simões, Steve O’Hagan, Enzhi Jin, Philip J. Day

**Affiliations:** 1 Division of Evolution and Genomic Sciences, Faculty of Biology, Medicine and Health, The University of Manchester, Manchester M13 9PL, UK; enhui.jin@postgrad.manchester.ac.uk (E.J.); maria.ritagildasilvasimoes@postgrad.manchester.ac.uk (M.R.G.d.S.S.); 2 School of Chemistry, Department of Chemistry, The University of Manchester, Manchester M13 9PL, UK; sohagan@manchester.ac.uk; 3 Division of Biosciences, Faculty of Life Sciences, University College London, London WC1E 6BT, UK; evelyn.jin.24@ucl.ac.uk; 4 Department of Medicine, University of Cape Town, Cape Town 7925, South Africa

**Keywords:** drug sensitiser, small molecule, PDAC, solid tumour, gemcitabine, NF-κB signalling, drug combination

## Abstract

**Background/Objectives:** Pancreatic ductal adenocarcinoma (PDAC) exhibits poor clinical response to gemcitabine, largely due to intrinsic and acquired mechanisms of chemoresistance. Identifying agents capable of enhancing gemcitabine efficacy without increasing cytotoxicity remains an unmet therapeutic need. Here, we characterise a small drug sensitiser molecule, B12, and evaluate its potential to sensitise PDAC cells to gemcitabine. **Methods:** Gemcitabine’s dose–response was assessed by MTT assay to determine IC_50_ values and dose-modifying factor (DMF). Phenotypic consequences of co-treatment were examined using colony formation and wound scratch assays. Mitochondrial membrane potential (JC-1) and apoptosis (Annexin V/PI) were measured using flow cytometry. Transcriptomic profiling was performed using mRNA-seq with differential expression analysis and pathway enrichment (KEGG/GSEA). NF-κB activity was assessed by nuclear and cytoplasmic fractionation of p65, and RT-qPCR validation of NF-κB associated target genes. **Results:** B12 alone displayed minimal cytotoxicity in the PANC-1 cell line and normal pancreatic ductal HPDE cells, yet shifted the gemcitabine dose–response curve in PANC-1 cells, reducing the IC_50_ and yielding a dose-modifying factor of 1.39. Functionally, B12 enhanced gemcitabine-induced suppression of colony formation and reduced wound closure relative to gemcitabine alone. The co-treatment also increased both mitochondrial depolarisation and apoptotic cell populations, with increased cell proliferation inhibition over time. Transcriptomic profiling identified a set of B12-associated genes downregulated both in B12-treated and B12 + gemcitabine conditions, including factors linked to growth, survival, inflammation, metabolism, and drug inactivation. Gene set enrichment analysis revealed negative enrichment of NF-κB associated pathways during B12 co-treatment. Consistently, nuclear-cytoplasmic fractionation showed that B12 reduced gemcitabine-induced nuclear accumulation of p65, accompanied by decreased expression of NF-κB associated targets such as *BCL2L1*, *CCL20*, *SLC2A1*, and *MAP3K14*. **Conclusions:** In PDAC cell models, B12 enhances gemcitabine cytotoxic response while displaying minimal intrinsic toxicity under the conditions tested. The sensitising phenotype is accompanied by increased apoptotic susceptibility and is associated with reduced NF-κB signalling at the pathway, transcript, and p65 nuclear localisation levels. However, to establish causality, the lack of sensitisation in HPDE cells will require further validation.

## 1. Introduction

Pancreatic ductal adenocarcinoma (PDAC) remains one of the most lethal malignancies, with a 5-year survival rate below 10% and minimal improvement in treatment outcome over the past several decades [1,2]. Gemcitabine continues to serve in first-line therapy, either alone or in combination regimens; however, clinical benefit is frequently limited by the rapid development of chemoresistance and dose-limiting toxicity associated with combination treatments [2,3]. Overcoming gemcitabine resistance while avoiding drug cytotoxic burden remains a most challenging issue in PDAC therapy [4].

Multiple mechanisms contribute to gemcitabine resistance, including diminished drug uptake or increased drug release, impaired activation by deoxycytidine kinase, increased metabolic inactivation by cytidine deaminase (CDA), and a wide array of pro-survival signalling pathways [5,6,7]. Among these, NF-κB activation is one of the most consistently implicated drivers of gemcitabine resistance [8,9]. Stress-induced NF-κB signalling promotes the expression of anti-apoptotic mediators such as *BCL-XL* and pro-inflammatory cytokines such as *CCL20*, collectively elevating the threshold for drug-induced apoptosis [10,11,12]. Pharmacologic or genetic inhibition of NF-κB has repeatedly been shown to restore gemcitabine responsiveness in PDAC models [13,14,15,16].

A drug sensitiser is defined as an agent with minimal intrinsic cytotoxicity that shifts the dose–response curve of a partner drug, quantified by a dose-modifying factor (DMF) greater than 1 [17,18,19,20]. Although widely applied in radiotherapy research, this concept is increasingly recognised as relevant to chemotherapy, as it directly captures the “dose-lowering” benefit that many combination drug regimens aim to achieve [21,22].

**Figure 1:**
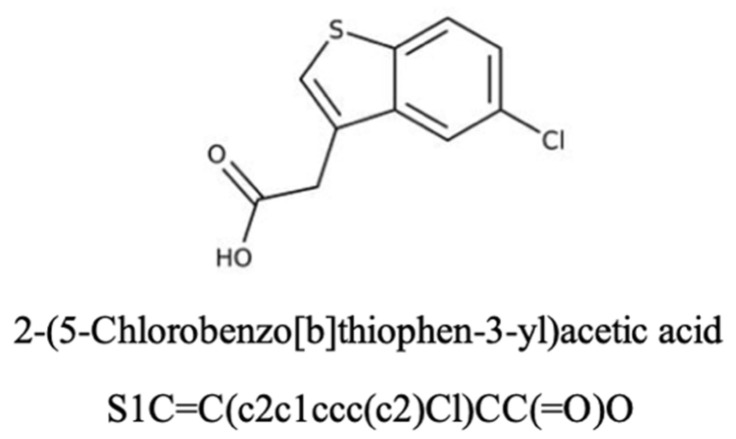
The chemical structure and name of B12 are shown with SMILES. The compound was identified from the Maybridge Fragment library, and screening details are described in a previous study [17].

Here, we investigate a small molecule, B12 (Figure 1), as a potential gemcitabine sensitiser in PDAC from Maybridge Fragment library (MBF) screening [17]. B12 was prioritised from the Maybridge Fragment (MBF) library screening as it showed minimal cytotoxicity on its own while improving gemcitabine response in our screening workflow. This fits the criteria of a sensitiser—reduce dosing without adding standalone toxicity. As an early-stage hit, this compound will allow for further structural modification to optimise efficacy. In silico SwissADME analysis was performed to predict B12’s pharmacokinetics and bioavailability. The compound presents an Abbott bioavailability score of 0.85, indicating a high probability of favourable oral bioavailability in humans. This metric is widely used in pharmacology for investigating properties essential for drug discovery, possible associated toxicities, and overall drug-likeness [23]. The high bioavailability score of B12 suggests its potential use as a drug-sensitising agent. Nevertheless, as this score is derived from a computational approach, further metabolic studies should be employed to further validate B12’s in vivo bioavailability. In addition, B12 was predicted not to be a substrate for the permeability glycoprotein (P-gp), a key member of the ATP-binding cassette efflux transporter family. This feature is particularly important in the context of intracellular drug availability as P-gp’s efflux activity has been strongly associated with the development of multidrug resistance in tumours [24]. There is currently no experimental data available regarding potential off-target effects of B12. However, SwissADME analysis identified B12 as a potential inhibitor of the cytochrome P450 CYP1A2, an isoenzyme with important roles in the metabolic clearance of drug compounds. Inhibition of these cytochromes can potentially increase the risk of pharmacokinetic drug–drug interactions due to reduced drug clearance and increased accumulation. Although this prediction does not correlate with intrinsic toxicity, it highlights a potential off-target effect of B12. Collectively, these computational predictions support the hypothesis of B12’s potential as a gemcitabine sensitiser. However, experimental validation is required to ensure suitable bioavailability and identify potential off-target effects.

We show that B12 selectively enhances gemcitabine cytotoxicity in PANC-1 cells without exerting independent toxicity or increasing the toxicity towards normal pancreatic ductal HPDE cells. Using viability assays, clonogenic and migration analyses, mitochondrial depolarisation, apoptosis markers, transcriptomic profiling, and NF-κB pathway analysis, we examine the cellular processes associated with this sensitising effect. Collectively, these findings identify B12 as a chemical sensitiser that reduces gemcitabine-induced NF-κB activation and enhances apoptotic susceptibility to lower doses of gemcitabine.

## 2. Results

### 2.1. B12 Sensitised Pancreatic Cancer Cells to Gemcitabine Treatment

**Figure 2:**
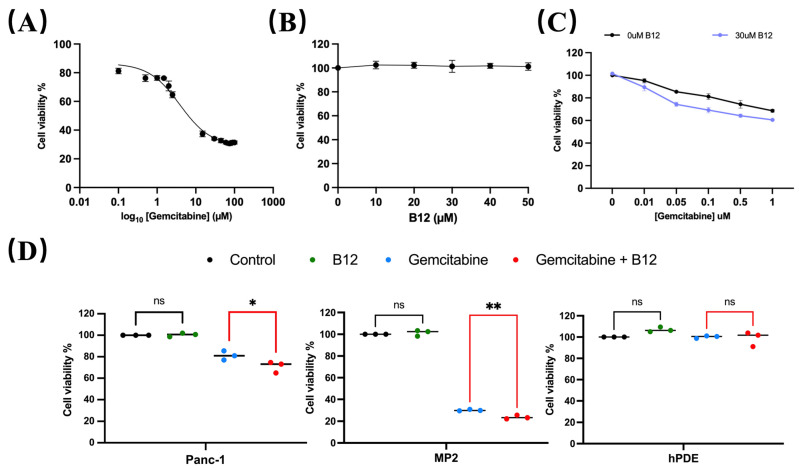
B12 reduces IC_50_ in PANC-1 and does not exert generalised toxicity to non-cancer cells. (**A**) PANC-1 cells were treated with various concentrations from 0 to 105 nM for 96 h, and the IC_50_ was measured via MTT assay. Nonlinear regression identified an IC_50_ of 4830 nM. Error bars represent the mean ± SEM. (**B**) B12 toxicity at 0–5 × 10^4^ nM was examined in PANC-1 cells for 96 h, showing no intrinsic cytotoxicity across tested concentrations. (**C**) Gemcitabine (10, 50, 100, 500, 1000 nM) was tested with or without 3 × 10^4^ nM B12 in PANC-1. IC_50_ reduced to 3460 nM with DMF of 1.39. (**D**) Viability of MiaPaca-2 (MP2) and HPDE cells with 100 nM gemcitabine alone or in combination with 3 × 10^4^ nM B12 was examined. A similar effect was observed in MP2, but minimal cytotoxicity was found in HPDE. Data was analysed using GraphPad Prism, with results expressed as mean ± SEM. Comparisons between treatment groups were evaluated using one-way ANOVA to determine statistical significance (ns = not significant; * *p* < 0.05; ** *p* < 0.01).

A concentration range of 0.1–100 μM gemcitabine was used to evaluate cytotoxicity in PANC-1 cells and determine the half-maximal inhibitory concentration (IC_50_). As shown in Figure 2A, the estimated IC_50_ for gemcitabine in PANC-1 was 4.8 μM, consistent with previous reports classifying PANC-1 as a gemcitabine-non-sensitive pancreatic cancer cell line that requires relatively high drug concentrations to achieve growth inhibition [25,26]. Within the experimental range, gemcitabine concentrations below IC_50_ produced limited growth inhibition after 96 h treatment, whereas a measurable viability suppression was observed at concentrations approaching the IC_50_. These operational dose ranges were therefore used to define relatively “non-responsive” and “responsive” in subsequent analyses. To assess whether B12 exhibited intrinsic cytotoxicity, PANC-1 cells were exposed to 0–50 μM B12. No reduction in cell viability was observed across the tested concentrations (Figure 2B), indicating that B12 alone does not impair cell survival under these conditions. We next examined whether B12 modulates gemcitabine sensitivity. Co-treatment with B12 resulted in a downward shift in the gemcitabine dose–response curve (Figure 2C) and a reduction in the IC_50_ from 4.83 μM (gemcitabine alone) to 3.46 μM (gemcitabine + B12). The corresponding DMF, calculated as the ratio of the two IC_50_ values, was 1.39. This suggests a measurable sensitisation of PANC-1 cells to gemcitabine. Notably, this IC_50_ shift occurred within the concentration range where PANC-1 cells exhibited limited response to gemcitabine, which indicates that B12 enhances gemcitabine efficacy within a transitional response window rather than simply amplifying high-dose cytotoxicity. To determine whether this sensitising effect was selective, additional cancerous and non-cancerous cell lines were evaluated. Human pancreatic cancerous cells—MiaPaca-2 (MP2), and non-malignant pancreatic ductal epithelial control cells—HPDE, were tested under the same conditions. Gemcitabine showed nearly no cytotoxicity for HPDE cells, whereas it showed significant enhancement of gemcitabine cytotoxicity for the two cancerous cell lines (Figure 2D), suggesting that B12 does not exert generalised toxicity in non-malignant pancreatic cells. PANC-1 cells were selected for further sensitisation mechanism study as a gemcitabine non-sensitive model. Studies have shown that the PANC-1 is resistant to gemcitabine treatment and has shown significant EMT features [27,28]. In addition, comparative analyses have revealed that PANC-1 presents significantly higher basal NF-κB levels than MiaPaCa-2 cells. This further supports the choice of using PANC-1 as a mechanistic model for studying NF-κB-mediated survival and chemoresistance [29].

### 2.2. Combination of B12 and Gemcitabine Reduced Cell Migration and Colony Formation

**Figure 3:**
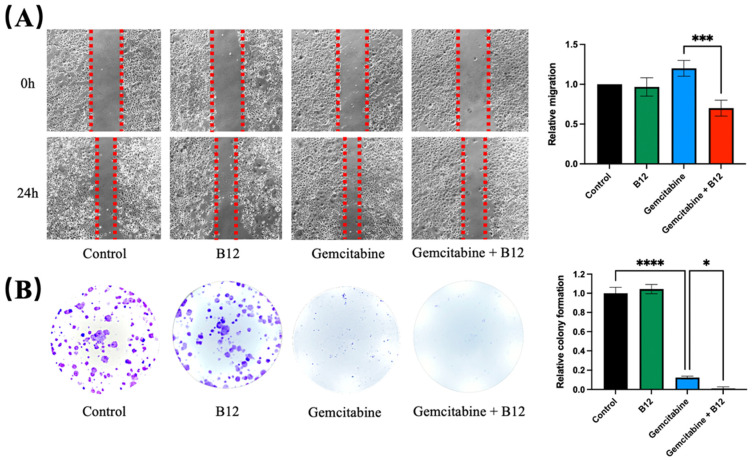
B12 + gemcitabine exhibits anti-clonogenicity and migration effect. (**A**) A wound scratch assay was performed on PANC-1 cells treated with B12, gemcitabine, and their combination over 24 h. Representative images of the wound and the recovering areas marked by red dotted lines were taken and analysed. (**B**) Colony formation assay was conducted to investigate the clonogenicity of each group after 48 h of treatment exposure. The number of colonies was counted and analysed after 7 days of recovery. Data were analysed using GraphPad Prism, with results expressed as mean ± SEM. Comparisons between treatment groups were evaluated using one-way ANOVA to determine statistical significance (* *p* < 0.05; *** *p* < 0.001, **** *p* < 0.0001).

Given that cell migration and clonogenicity are key phenotypic features associated with cancer progression, the potential impact of B12 on these processes was next examined [30]. A wound-scratch assay was performed to assess changes in migration. As shown in Figure 3A, PANC-1 cells treated with gemcitabine alone resulted in a marginally faster wound closure, whereas B12 alone had no effect and resembled the control. Previous reports have shown that gemcitabine treatment can enrich for EMT-like, more migratory pancreatic cancer cells and increase wound closure in surviving populations, which align with the observed accelerated wound closure upon gemcitabine exposure in PANC-1 cells [5]. Notably, the combined B12 and gemcitabine treatment resulted in a significant reduction in migration, with slower wound closure compared to gemcitabine alone. (1.2, 0.7, *p* = 0.0007) Given that wound closure reflects both migratory behaviour and population dynamics of surviving cells, the faster closure observed with gemcitabine is likely due to enrichment of treatment-resistant, EMT-like subpopulations rather than an increase in overall migratory capacity [5,31]. B12 co-treatment with gemcitabine reduced wound closure relative to gemcitabine alone, indicating suppression of gemcitabine-induced motility or an overall enrichment of less migratory subpopulations (Figure 3A). To determine whether long-term proliferation potential was affected, a clonogenic assay was conducted. As expected, gemcitabine substantially reduced colony formation relative to the untreated control (Figure 3B) [32]. However, co-treatment with B12 produced an even greater decrease in clonogenic survival compared with gemcitabine alone, demonstrating that B12 enhances the suppression of long-term growth imposed by gemcitabine. (0.12, 0.01, *p* = 0.04)

### 2.3. B12 and Gemcitabine Combination Induced Apoptosis and Reduced Cell Proliferation

**Figure 4:**
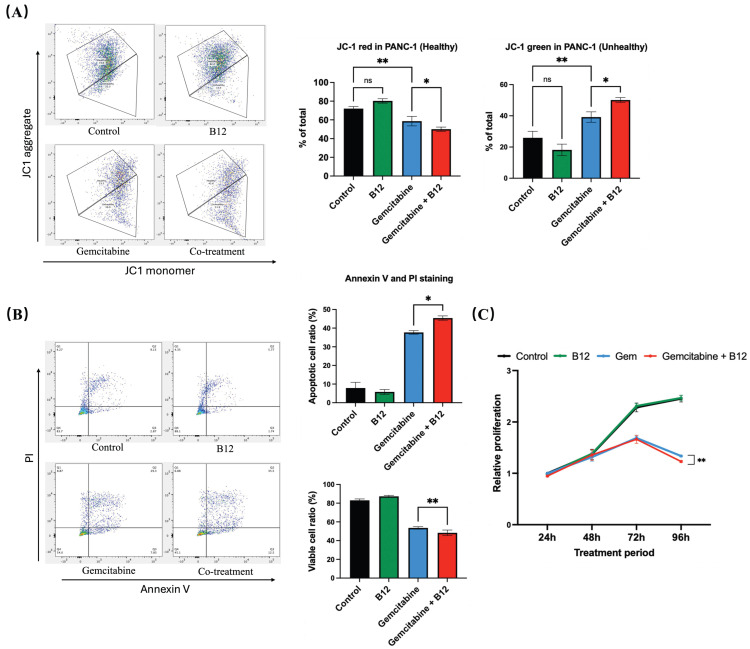
B12 + gemcitabine enhanced cell apoptosis and inhibited cell proliferation. (**A**) JC-1 mitochondrial membrane potential analysis after 48 h treatment. Control and B12-treated cells displayed high red fluorescence, indicating polarised mitochondria. Gemcitabine increased the green signal, reflecting depolarisation, whereas B12 + gemcitabine further enhanced the shift toward depolarised mitochondrial membranes. (**B**) Annexin V/PI analysis. Co-treatment with B12 and gemcitabine increased the proportion of apoptotic cells compared with gemcitabine alone. (**C**) Time-course MTT assay (24–96 h) showed that B12 alone did not affect cell viability at any time point. Gemcitabine progressively inhibited cell growth beginning at 72 h, and co-treatment with B12 produced significant suppression of cell growth at 96 h. Data were analysed using GraphPad Prism, with results expressed as mean ± SEM. Comparisons between treatment groups were evaluated using one-way ANOVA to determine statistical significance (ns = not significant; * *p* < 0.05; ** *p* < 0.01).

Mitochondrial membrane potential was evaluated using fluorescent probe JC-1 staining following 48 h of treatment (Figure 4A) [33]. In control and B12-treated cells, the proportion of JC-1 red fluorescence (indicative of polarised, healthy mitochondria) remained high, with no significant difference between the two groups. Gemcitabine alone led to a reduction in red signal and a corresponding increase in JC-1 green fluorescence, reflecting a shift toward mitochondrial depolarisation. Notably, the B12 + gemcitabine co-treatment further enhanced green fluorescence compared with gemcitabine alone by 11% with *p* = 0.016, indicating a more significant loss of mitochondrial membrane potential. As mitochondrial depolarisation is an early hallmark of apoptosis, these findings suggest that B12 enhances gemcitabine-induced apoptotic mitochondrial dysfunction [34].

To examine whether apoptosis contributed to the enhanced cytotoxicity, Annexin V and PI double staining was performed (Figure 4B). The combination of B12 with gemcitabine further increased the apoptotic cell ratio relative to gemcitabine alone by 7.7% with *p* = 0.047, indicating that B12 increases gemcitabine-induced apoptotic signalling.

To further characterise the antiproliferative effects of B12, a time-course MTT assay was conducted from 24 to 96 h (Figure 4C). B12 alone showed no difference compared with the untreated control at any of the tested timepoints, indicating a lack of intrinsic cytotoxicity under these conditions. From 72 h onwards, gemcitabine progressively reduced cell growth, consistent with the reported doubling time of PANC-1 cells of approximately 55 h. Notably, co-treatment of B12 and gemcitabine resulted in a more significant suppression of proliferation relative to gemcitabine alone by 0.1-fold change with *p* = 0.008 at 96 h.

### 2.4. Pathways Associated with B12 Treatment Revealed by Transcriptomic Profiling

**Figure 5:**
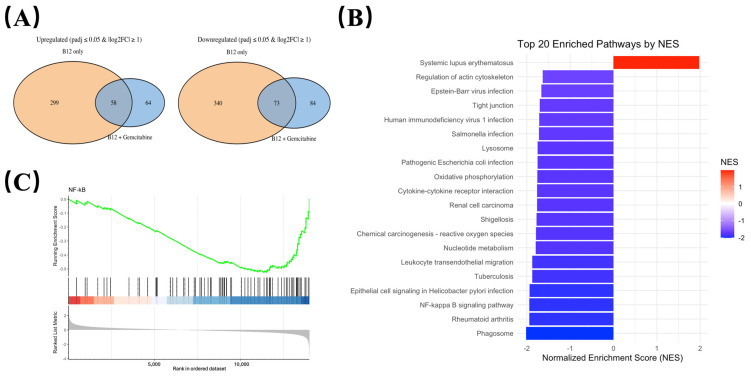
Transcriptomic profiling identifies NF-κB signalling pathway is suppressed by B12. (**A**) Overlap analysis of differentially expressed genes shared between the B12 vs. control is shown as orange, and B12 + gemcitabine vs. gemcitabine as blue. Overlap genes consistently altered in both conditions were defined as B12-associated transcriptional responses. (**B**) KEGG pathway enrichment analysis of genes regulated by B12 in the co-treatment group compared to the gemcitabine group, with normalised enrichment scores (NES) indicating the up-/down-regulation. (**C**) Gene Set Enrichment Analysis (GSEA) comparing B12 + gemcitabine vs. gemcitabine alone. The NF-κB gene set displayed a negative normalised enrichment score (in blue), indicating coordinated downregulation of NF-κB-associated genes during co-treatment.

To identify transcriptional changes that were consistently associated with B12 exposure, differential expression was compared between: (i) B12 vs. control, and (ii) B12 + gemcitabine vs. gemcitabine alone (Illumina, Novaseq). Gene expressions that significantly changed in the same direction (either upregulated or downregulated) in both comparisons were defined as B12-associated overlap genes, representing transcriptional responses attributable to B12 regardless of the presence of gemcitabine (Figure 5A).

Using this criterion, we identified a set of downregulated overlap genes enriched for growth, survival, and inflammatory-related pathways (Table 1). These included genes such as *AREG*, *CCL20*, and *MAP3K14* (*NIK*), all showing consistent reductions in both comparisons. The reduction in *MAP3K14* mRNA suggests a potential reduction in upstream NF-κB regulatory elements.

(1) Growth factor and NF-κB-linked survival signalling

Several downregulated overlap genes were associated with growth and survival pathways, including *AREG* (an EGFR ligand), *CCL20* (a RelA-dependent chemokine), *MAP3K14/NIK* (a kinase driving alternative NF-κB activation), and *SFN/14-3-3σ*, all of which have reported roles in promoting proliferation, invasive traits, or therapy resistance in PDAC [35,36,37,38]. The coordinated suppression of these pro-survival factors aligns with the observed decrease in nuclear p65 and the enhanced apoptotic response in the B12-gemcitabine group, indicating that B12 may reduce NF-κB associated survival signalling, thereby lowering resistance to cytotoxic stress.

(2) Metabolic survival

**Figure 6:**
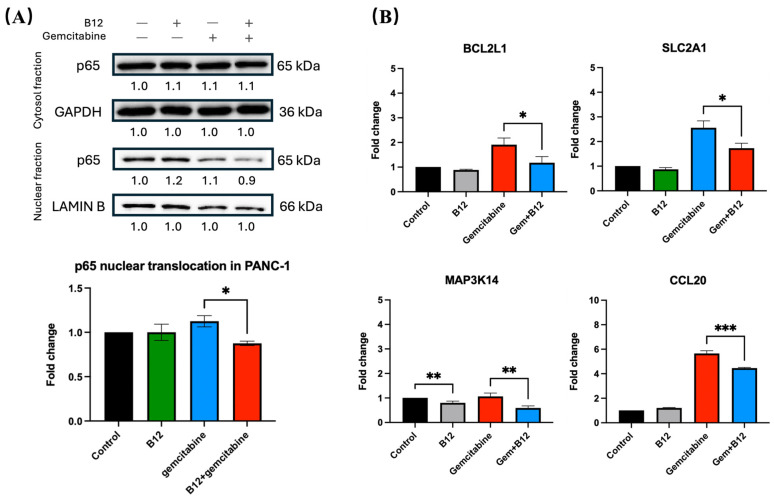
B12 reduces nuclear translocation of p65 and downregulates NF-κB associated genes. (**A**) Nuclear and cytoplasmic fractionation analysis of p65. Gemcitabine increased nuclear p65 levels, whereas co-treatment with B12 reduced gemcitabine-induced nuclear translocation without altering cytoplasmic p65 expression. (**B**) RT-qPCR analysis of representative NF-κB associated target genes identified in RNA-seq. Co-treatment significantly reduced expression of *BCL2L1*, *CCL20*, *SLC2A1* (*GLUT1*), and *MAP3K14* (*NIK*) compared with gemcitabine alone. Data were analysed using GraphPad Prism, with results expressed as mean ± SEM. Comparisons between treatment groups were evaluated using one-way ANOVA to determine statistical significance (* *p* < 0.05; ** *p* < 0.01; *** *p* < 0.001).

Among the downregulated overlap genes, *SLC2A1* (*GLUT1*)—a key glucose transporter linked to glycolytic metabolism and poor prognosis in PDAC—was notably reduced and validated by RTqPCR analyses (results shown in Figure 6) [39]. Reduced *GLUT1* expression suggests decreased metabolic support for cell survival, which is consistent with the increased apoptotic susceptibility detected in functional assays [40]. However, functional metabolic assessments would be required to establish whether B12 affects glycolysis directly.

(3) Drug metabolism

The overlap region also included cytidine deaminase (CDA), a validated determinant of gemcitabine inactivation and chemoresistance in PDAC. Lower *CDA* expression may reduce gemcitabine catabolism and thereby increase intracellular drug activity, offering a potential pharmacologic basis for the observed sensitisation effect [41].

To assess pathway-level changes associated with B12 exposure, KEGG enrichment analysis was performed using the overlap gene set shared between B12 alone and B12–gemcitabine treatment. As shown in Figure 5B, most enriched pathways exhibited negative normalised enrichment scores (NES), indicating an overall downregulation trend. Several immune-related categories appeared among the top-ranked pathways, which is expected given the broad involvement of NF-κB in inflammatory transcriptional programs. Notably, the NF-κB signalling pathway was negatively enriched, suggesting that genes associated with NF-κB activity were suppressed following B12 exposure. The role of NF-κB is well-established in driving cell survival and treatment resistance in PDAC, and the observed enrichment pattern prompted further investigation of NF-κB signalling dynamics.

Gene Set Enrichment Analysis (GSEA) was performed comparing the B12 + gemcitabine group to gemcitabine alone. As shown in Figure 5C, the NF-κB gene set displayed a left-shifted running enrichment curve with a negative enrichment score, indicating that NF-κB-associated genes were collectively downregulated under co-treatment conditions.

### 2.5. Co-Treatment with B12 Can Reduce p65 Nuclear Translocation and NFkB Activation

To determine whether the sensitising effect of B12 involves modulation of NF-κB signalling, nuclear and cytoplasmic fractions of PANC-1 cells were analysed for p65 (RELA) protein expression. As shown in Figure 6A, gemcitabine alone led to an increase in nuclear p65, consistent with NF-κB activation following chemotherapeutic stress. In contrast, co-treatment with B12 substantially reduced nuclear p65 levels relative to gemcitabine alone by 0.3-fold change with *p* = 0.04, while cytoplasmic p65 remained unchanged, indicating that B12 interferes with gemcitabine-induced NF-κB nuclear translocation. This pattern indicates altered subcellular distribution rather than changes in total p65 abundance.

Given that NF-κB transcriptionally regulates multiple anti-apoptotic and pro-survival genes, we next examined representative downstream targets identified from the mRNA-seq dataset (Figure 6B) [42]. RT-qPCR analysis demonstrated that co-treatment with B12 and gemcitabine significantly reduced mRNA expression of *BCL2L1* compared with gemcitabine alone by 0.7 with *p* = 0.03. Because *BCL-XL* promotes mitochondrial survival and apoptosis resistance, its suppression will lead to enhanced apoptotic response in co-treated cells [43,44,45]. Similarly, the NF-κB-associated chemokine *CCL20* was significantly downregulated in the combination group relative to gemcitabine by 1.1 with *p* = 0.0009, consistent with prior evidence linking *CCL20* to NF-κB-dependent survival and therapy resistance in pancreatic cancer [46]. In addition, the metabolic transporter *SLC2A1* (*GLUT1*), which supports glycolytic survival, was decreased upon co-treatment by 0.9 with *p* = 0.01, suggesting that B12 may further weaken cellular metabolic fitness under gemcitabine exposure [47,48]. Finally, expression of *MAP3K14* (*NIK*), a kinase driving alternative NF-κB signalling, was reduced in the co-treatment condition by 0.5 with *p* = 0.008, supporting a broader suppression of NF-κB activation upstream of p65 nuclear translocation [15]. Despite a significant decrease in *MAP3K14* expression in the B12 group of 0.2 with *p* = 0.009, the expression of all other genes showed no statistically significant differences compared with the control.

## 3. Discussion

In this study, we identify chemical fragment B12 as a small molecule sensitiser that enhances the cytotoxic effects of gemcitabine in PANC-1 and MP2 pancreatic cancer cells. B12 displayed minimal intrinsic toxicity yet significantly shifted the gemcitabine dose–response curve, consistent with the classical definition of a chemical sensitiser. To contextualise the magnitude of B12-mediated sensitisation, we calculated a dose-modifying factor (DMF) of 1.39. Although clinical studies of gemcitabine combination regimes with compounds such as nap-paclitaxel and capecitabine rely on overall or progression-free survival instead of sensitisation metrics, preclinical studies in PDAC models provide comparable insights into B12’s relative effect as a sensitizer [49,50]. As an example, Curcumin has been shown to sensitise the previously resistant Panc-1 cells to radiation treatment with sensitisation enhancement ratios (SER) varying from ~1.53 to ~1.93 in a concentration range of 10–12 uM [51]. A different study reported that the use of pharmacological ascorbate in combination with gemcitabine resulted in higher levels of cytotoxicity in several different pancreatic cell lines. In Panc1 cells, the combination treatment produced a drug response index (DRI) varying from 1.4 to 3.5 [52]. These comparisons place B12 within the range of other reported gemcitabine sensitisers.

Although this sensitising effect was not observed in HPDE cells under the tested conditions, this finding should not be interpreted as tumour selectivity. Several alternative explanations may account for the differential responses between HPDE and PANC-1 cells. First, differences in proliferation kinetics may influence apparent drug sensitivity during extended treatment periods. HPDE cells exhibit near-normal epithelial growth rate, which may affect cumulative cytotoxic readouts following gemcitabine exposure. Second, activation of NF-κB signalling is frequently reported in pancreatic ductal adenocarcinoma and has been linked to oncogenic KRAS-driven survival pathways [43]. In contrast, non-transformed ductal epithelial cells are not generally characterised by sustained NF-κB activation. However, basal NF-κB activity was not directly quantified in HPDE and PANC-1 cells in the present study, and therefore, differential baseline signalling remains a reasonable but untested explanation. Finally, cellular uptake and metabolism of gemcitabine may differ between cancerous and non-cancerous cells. Gemcitabine efficacy depends on nucleoside transporters and metabolic enzymes, and variations in these processes could alter intracellular drug exposure and modulate the apparent impact of B12 co-treatment [53]. As these factors were not measured here, further investigation is required to conclude as tumour selective sensitiser.

Across multiple phenotypic assays, the combination further reduced clonogenic capacity, suppressed migration, and elevated apoptosis levels. These findings support a model in which B12 promotes an apoptosis-prone state under gemcitabine treatment rather than exerting direct cytotoxicity. RNA-seq analysis revealed transcriptional changes associated with B12 exposure, particularly the downregulation of growth and survival-related pathways. Notably, several genes linked to NF-κB signalling—including AREG, *CCL20*, and *MAP3K14*—were significantly reduced. GSEA demonstrated negative enrichment of NF-κB gene sets during B12-gemcitabine co-treatment, and nuclear-cytoplasmic fractionation indicated reduced gemcitabine-induced nuclear accumulation of p65. Taken together, these findings are consistent with reduced NF-κB pathway activity during B12 co-treatment. In addition, B12 treatment was associated with downregulation of metabolic and mitochondria-related gene modules and increased JC-1 green fluorescence, consistent with weakened mitochondrial integrity under stress. However, because mitochondrial depolarisation can arise during apoptosis, further metabolic flux analyses would be required to determine whether B12 directly modulates oxidative phosphorylation [54]. Thus, these data are best interpreted as supporting an association between B12 treatment and increased early apoptosis, rather than a primary mechanistic driver.

Several limitations should be noted. Firstly, a functional investigation is needed. While consistent with reduced NF-κB activity, the current data do not establish a direct causal relationship between NF-κB suppression and sensitisation. Rescue experiments targeting individual NF-κB components or downstream genes would clarify this relationship. Secondly, although B12-associated changes in metabolic gene expression were observed, metabolic flux analyses (e.g., Seahorse) were not performed. Thirdly, NF-κB is found to be relevant in PDAC resistance and gemcitabine response, but PDAC models vary substantially. Therefore, pathway validation in additional PDAC models, including MP2 cells, will be required to assess the generalizability of these findings. And the absence of sensitisation in HPDE cells needs direct comparative analyses to define the determinants of B12-mediated selective sensitisation. Finally, the key limitation of this study is the absence of in vivo validation. Although the in vitro assays used in this study provided significant insights into B12-mediated gemcitabine sensitisation, these do not reflect the complexity of systemic drug exposure, tumour heterogeneity, and toxicity. That being said, in vivo approaches will be essential to further validate B12’s activity as a sensitising agent and to investigate any potential associated toxicities.

In summary, our findings identify B12 as a non-cytotoxic agent that enhances gemcitabine efficacy in PANC-1 cells by reducing NF-κB activation and promoting apoptotic susceptibility. These results highlight a potential strategy to improve gemcitabine responsiveness without increasing treatment toxicity and provide a foundation for further mechanistic and translational investigation.

## 4. Materials and Methods

### 4.1. Cell Lines and Reagents

Human pancreatic ductal adenocarcinoma (PDAC) cell lines PANC-1, MiaPaca-2, and human pancreatic duct epithelial cells (HPDE) were obtained from ATCC. Cells were cultured under the following conditions: PANC-1 and MiaPaca-2: DMEM supplemented with 10% foetal bovine serum (FBS) and 1% penicillin-streptomycin. HPDE cells were cultured in keratinocyte-SFM supplemented with EGF and bovine pituitary extract (Thermo Fisher, Waltham, MA, USA), following the supplier’s recommendations. All cell lines were maintained at 37 °C with 5% CO_2_. Gemcitabine was dissolved in sterile water. Compound B12 was dissolved in DMSO (final DMSO ≤ 0.05%). B12 was screened from the MBF library, and the protocol is described in a previous study [17]. Both drugs were freshly diluted into undiluted medium before use.

### 4.2. Cell Viability Assay (MTT)

Cell viability was assessed using the MTT assay [55]. Cells were seeded at 5 × 10^3^ cells/well in 96-well plates. After drug exposure, MTT reagent (1 mg/mL MTT powder in PBS) was added and incubated for 3 h. Absorbance was measured at 570 nm. Dose–response curves and IC_50_ values were fitted using nonlinear regression in GraphPad Prism v10.5.0.

### 4.3. Colony Formation Assay

Cells (5 × 10^2^/well) were treated with gemcitabine, B12, or both for 48 h, followed by drug-free culture for 7–10 days. Colonies were fixed with 4% paraformaldehyde, stained with 1% crystal violet, and quantified using ImageJ.

### 4.4. Wound-Scratching Migration Assay

Confluent monolayers were scratched with a pipette tip, washed to remove debris, and treated with indicated drug conditions in serum-free medium. Images were collected at 0 h and 24 h. Wound area was quantified using QuPath v0.501.

### 4.5. JC-1 Mitochondrial Membrane Potential Assay

Cells were treated for 48 h and incubated with JC-1 dye according to the manufacturer’s protocol (Cell Signalling, Danvers, MA, USA). Samples were analysed by flow cytometry (BD), and the red/green fluorescence ratio was calculated via FlowJo 10.4.

### 4.6. RNA Sequencing and Bioinformatic Analysis

Total RNA was extracted using the Qiagen miRNeasy kit. RNA sequencing was performed using Illumina (Novogene, Bejing, China). Differential expression analysis was conducted using DESeq2, applying Benjamini–Hochberg false discovery rate (FDR) correction. Significance cutoff: adjusted *p*-value (padj) < 0.05 and |log_2_FC| > 1. Pathway enrichment analysis was performed using KEGG and clusterProfiler in R. Gene Set Enrichment Analysis (GSEA) was used to evaluate curated gene sets, including NF-κB related signatures.

### 4.7. Nuclear–Cytoplasmic Fractionation and Western Blotting

Nuclear and cytoplasmic fractions were prepared using standard extraction buffers. Proteins were quantified by BCA assay and resolved via SDS-PAGE. Primary antibodies (Proteintech, Rosemont, IL, USA) included p65 (RELA), GAPDH (cytoplasmic control), and Lamin B1 (nuclear control). Signals were detected using ECL and quantified using ImageJ 1.54g.

### 4.8. Real-Time Quantitative PCR (RT-qPCR)

RNA was reverse-transcribed and analysed by qPCR on a Roche 480 Light Cycler using SYBR Green (Roche, Basel, Switzerland). Gene expression was normalised to *ACTB* using the ΔΔCt method [56]. All primer sequences are detailed in Table 2.

### 4.9. Statistical Analysis

All experiments were performed with at least three independent biological replicates. Two-group comparisons: unpaired two-tailed Student’s *t*-test. Multiple-group comparisons: one-way ANOVA followed by Tukey’s post hoc test. Data are presented as mean ± SEM. *p* < 0.05 was considered statistically significant.

## Figures and Tables

**Table 1:** Downregulated B12-associated overlap genes and their functional categories.

Category	Representative Genes	Function
Growth/NF-κB	*AREG*, *CCL20*, *MAP3K14*, *SFN*	Survival signalling, resistance
Metabolic survival	*SLC2A1*	Glycolytic support, poor prognosis
Drug metabolism	*CDA*	Gemcitabine resistance

**Table 2:** Primer sequences of target genes and housekeeping gene ACTB.

Gene	Forward Sequence	Reverse Sequence
*CCL20*	AAG TTG TCT GTG TGC GCA AAT CC	CCA TTC CAG AAA AGC CAC AGT TTT
*BCL2L1*	CCCGCGACTCCTGATTCATT	AGTCTACTTCCTCTGTGATGTTGT
*MAP3K14*	CCA GAG GTG ATA CGG AAT GAA CC	TGG AAG GTG GAG GCT GTT GCT T
*SLC2A1*	TTG CAG GCT TCT CCA ACT GGA C	CAG AAC CAG GAG CAC AGT GAA G
*ACTB*	ATT GGC AAT GAG CGG TTC	GGA TGC CAC AGG ACT CCA T

## Data Availability

The original contributions presented in this study are included in the article. Further inquiries can be directed to the corresponding author.

## Abbreviations

PDAC	Pancreatic ductal adenocarcinoma
CDA	Cytidine deaminase
MBF	Maybridge Fragment
MP2	MiaPaca-2
FBS	Foetal bovine serum
DRI	Drug response index
